# Clinical profile analysis and nomogram for predicting in-hospital mortality among elderly severe community-acquired pneumonia patients: a retrospective cohort study

**DOI:** 10.1186/s12890-024-02852-x

**Published:** 2024-01-17

**Authors:** Chang Wei, Xinyu Wang, Dingxiu He, Dong Huang, Yue’an Zhao, Xinyuan Wang, Zong’an Liang, Linjing Gong

**Affiliations:** 1 Department of Respiratory and Critical Care Medicine, West China Hospital, Sichuan University, No. 37 Guoxue Alley, 610041 Chengdu, Sichuan, Sichuan China; 2 Department of Emergency Medicine, The People’s Hospital of Deyang, Deyang, Sichuan China; 3 Department of Orthopaedics, West China Hospital, Sichuan University, Chengdu, Sichuan China

**Keywords:** Severe community-acquired pneumonia (SCAP), The elderly, Risk factors, Nomogram, In-hospital mortality

## Abstract

**Background:**

Severe community-acquired pneumonia is one of the most lethal forms of CAP with high mortality. For rapid and accurate decisions, we developed a mortality prediction model specifically tailored for elderly SCAP patients.

**Methods:**

The retrospective study included 2365 elderly patients. To construct and validate the nomogram, we randomly divided the patients into training and testing cohorts in a 70% versus 30% ratio. The primary outcome was in-hospital mortality. Univariate and multivariate logistic regression analyses were used in the training cohort to identify independent risk factors. The robustness of this model was assessed using the C index, ROC and AUC. DCA was employed to evaluate the predictive accuracy of the model.

**Results:**

Six factors were used as independent risk factors for in-hospital mortality to construct the prediction model, including age, the use of vasopressor, chronic renal disease, neutrophil, platelet, and BUN. The C index was 0.743 (95% CI 0.719–0.768) in the training cohort and 0.731 (95% CI 0.694–0.768) in the testing cohort. The ROC curves and AUC for the training cohort and testing cohort (AUC = 0.742 vs. 0.728) indicated a robust discrimination. And the calibration plots showed a consistency between the prediction model probabilities and observed probabilities. Then, the DCA demonstrated great clinical practicality.

**Conclusions:**

The nomogram incorporated six risk factors, including age, the use of vasopressor, chronic renal disease, neutrophil, platelet and BUN, which had great predictive accuracy and robustness, while also demonstrating clinical practicality at ICU admission.

## Introduction

Community-acquired pneumonia (CAP) is an acute infectious disease affecting the lung parenchyma and is acquired outside the hospital [1]. Although CAP is one of the leading causes of mortality in immunocompetent and immunocompromised patients, it is still easily neglected [2]. The Global Burden of Diseases, Injuries, and Risk Factors Study [3] revealed that in 2016, 336.5 million cases of lower respiratory tract infection were recorded, resulting in an incidence rate of 32.2 per 100,000 people worldwide. Advanced age, chronic lung disease, chronic heart disease, cardiovascular disease, diabetes mellitus, malnutrition, viral respiratory tract infections, immunocompromising conditions, and lifestyle factors such as smoking and excessive alcohol consumption were the factors that increased the risk of community-acquired pneumonia [4]. Severe community-acquired pneumonia (SCAP) is one of the most lethal forms of CAP with high mortality. Septic shock and respiratory failure are the most serious complications of SCAP, characterized by especially life-threatening. Intensive care unit (ICU) care is generally required [2, 5]. It has been reported that the one-year mortality of all CAP inpatients is approximately 30%, while it is around 50% in ICU CAP patients [4].

In elderly patients, impaired gag reflex, decreased mucociliary function, damaged immunity, impaired febrile response, and cardiopulmonary dysfunction contribute to an increased susceptibility to developing CAP [6]. Risk factors that predisposed the elderly to pneumonia included comorbid conditions, organ dysfunction, nutritional status, alcohol consumption and smoking [7]. The incidence of the disease was higher among elderly individuals, with a rate of 63.0/10,000 person-years in those aged 65–79 and increasing to 164.3/10,000 person-years after the age of 80 [8, 9]. SCAP occurs more frequently in those with comorbidities [10, 11]. All comorbidities were more frequent in the elderly group [12]. Compared to younger patients, elderly people might exhibit less prominent symptoms due to associated comorbidity or impaired immune systems. Consequently, pneumonia in elderly patients was characterized by increased mortality and morbidity compared to their younger counterparts [6].

There were rules used to determine the severity and prognosis prediction of CAP and to guide treatment. Both the pneumonia severity index (PSI) and CURB-65 were developed as prognostic models based on demographic characteristics and clinical data to predict 30-day mortality. Compared to the PSI, CURB-65 was rarely thought to be effective as clinical evidence in the site of care [2, 6]. Sepsis-3 is a new definition about the life-threatening organ dysfunction caused by a dysregulated host response to infection, in order to enable early recognition of critically ill patients and thereby improve their outcomes [13]. However, their predictability for elderly patients needs to be improved [14, 15].

As the population ages, the incidence is expected to rise. Therefore, it will become a severe problem for our society. To achieve a rapid and accurate decision for high-risk patients, our study aimed to develop a mortality prediction model specifically targeting elderly SCAP patients. This model could help clinicians rapidly recognize high-risk patients, so that they can receive adequate attention and treatment.

## Methods

### Study design and cohort

We performed a retrospective, observational, cohort study. The study was conducted in the medical ICU at West China Hospital in Chengdu, Sichuan Province from September, 2011 to September 2019 and was approved by the West China Hospital of Sichuan University Biomedical Research Ethics Committee (No. 2021 − 828).

The requirement for obtaining informed consent in this analysis was waived due to the retrospective noninterventional design. To construct and validate the nomogram, we randomly divided the patients in one database into two cohorts, the training and testing cohorts, in a 70% versus 30% ratio to ensure comparability between the two cohorts.

CAP was diagnosed if the onset occurred within 48 h after admission or before admission. SCAP was defined as meeting at least 1 major criterion: (1) septic shock with need for vasopressors; (2) respiratory failure requiring mechanical ventilation; or at least 3 minor criteria: (1) respiratory rate ≥ 30 breaths/min; (2) PaO_2_/FiO_2_ ratio ≤ 250; (3) multi-lobar infiltrates; (4) confusion/ disorientation; (5) blood urea nitrogen level ≥ 20 mg/dL; (6) white blood cell count < 100,000/µL; (7) core temperature < 36℃; and (8) hypertension requiring aggressive fluid resuscitation), according to the Infectious Diseases Society of America (IDSA)/American Thoracic Society (ATS) guidelines [16]. The elderly patients were at least 65 years old.

The exclusion criteria were as follows: (1) not elderly (< 65 years old), (2) severe immunosuppression: autoimmune diseases, human immunodeficiency virus infection, chemotherapy, or other immunosuppressive therapy, (3) residents of long-term care facilities and/or nursing homes, (4) repeated admission, (5) hospital acquired pneumonia, (6) discharged within 24 h of admission, and (7) incomplete data. All patients received standard care and therapy according to the CAP guidelines.

### Study outcomes and measurements

Clinical data of patients were collected within 24 h after admission to the ICU from electronic medical records. The data encompassed demographic characteristics, comorbidities, vital signs, hematological indicators, biochemical parameters, inflammatory markers, coagulation indicators and other laboratory tests. In the case of repeated laboratory tests within the first 24 h of admission, we chose the initial values for analysis. Two trained respiratory clinicians reviewed the medical records with standardized data collection forms. Any controversy was resolved by team discussion. All patient data were anonymized. The follow-up ended at discharge. The primary outcome was in-hospital death, and the secondary outcomes were ICU mortality, 7-day, 14-day and 28-day mortality after the diagnosis of SCAP.

### Statistical analysis

All statistical analyses and graphs were performed by SPSS for Windows (Version 25.0, Chicago, IL, USA) and R software (Version 4.1.1, https://www.R-project. org/). Continuous variables are described as medians [interquartile ranges (IQRs), 25–75%] or means ± standard deviations (SDs), while categorical variables are described as frequencies. ‘p ≤ 0.05’ was considered statistically significant. Demographic and clinical characteristics were compared between the survival group and dead group, as well as between the training group and testing group using Student’s t-test, Mann–Whitney U test, or chi-square test where appropriate. Univariate logistic regression analysis was initially conducted to find the potential variables related to in-hospital mortality within the training cohort (*p* < 0.05). The results are described as ORs with corresponding 95% confidence intervals (CIs). The variables above were enrolled in the next multivariate logistic regression analysis to identify the independent risk factors for hospital mortality, according to which the nomogram for the hospital mortality prediction model was constructed. Then, the robustness of this model was assessed using the concordance index (C index) and area under the receiver operating characteristic curve (ROC and AUC). Finally, calibration curve and decision curve analysis (DCA) were employed to evaluate the predictive accuracy of hospital mortality in elderly SCAP patients.

.

## Results

### Clinical characteristics of elderly SCAP patients

**Fig. 1:**
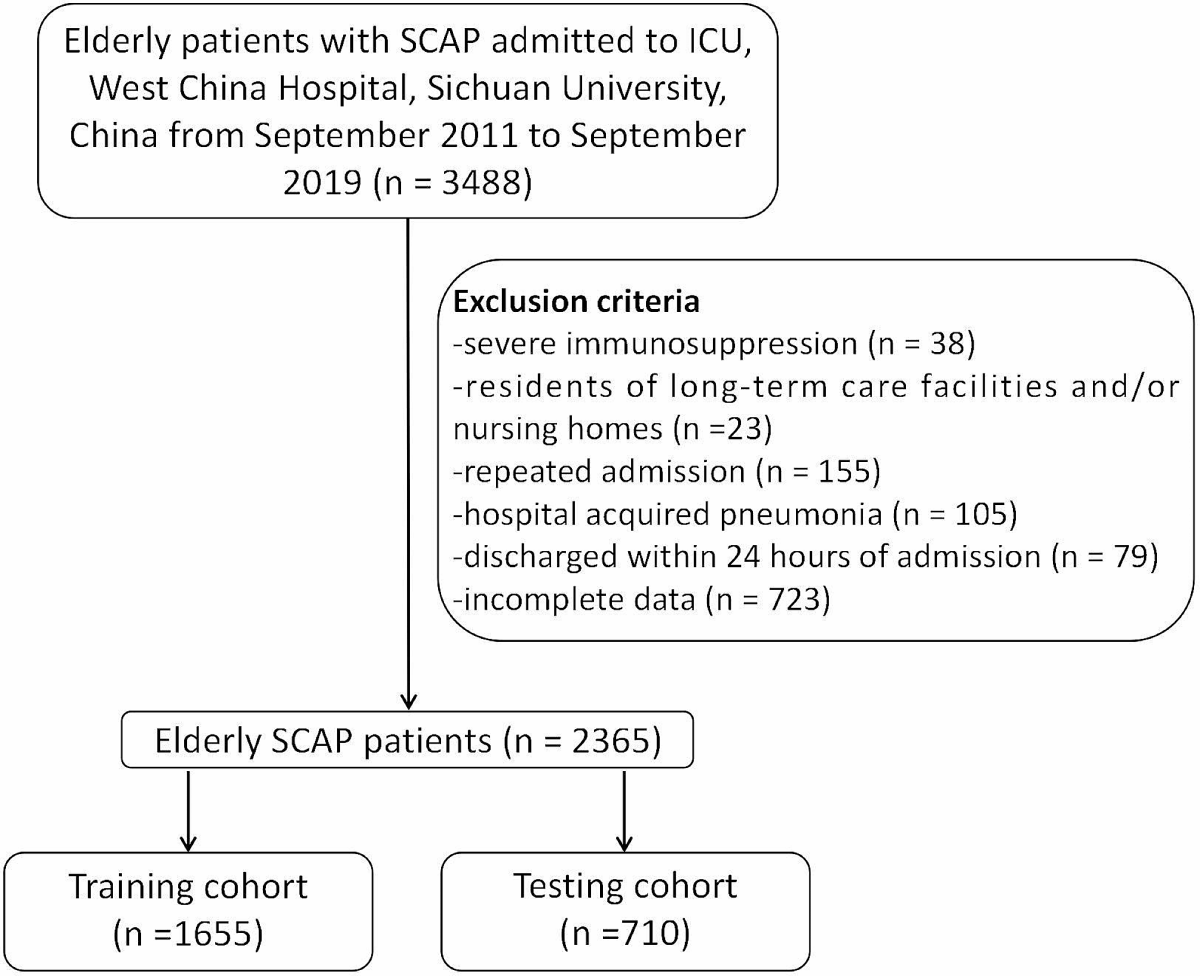
Flow chart of the present study

**Fig. 2:**
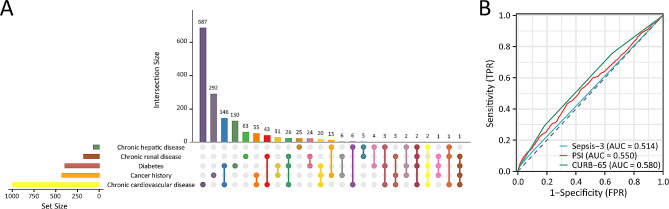
**A** The common coexisting medical conditions in elderly SCAP patients. **B** The ROC curve for PSI, CURB-65, and Sepsis-3

There were 3488 elderly SCAP patients enrolled in this study. People suffering from severe immunosuppression or hospital-acquired pneumonia, and people who were residents of long-term care facilities and/or nursing homes, had repeated admissions, were discharged within 24 h of admission, or had incomplete data were excluded (Fig. 1). Finally, a total of 2365 patients were enrolled in the subsequent analysis, which contained 1529 (64.7%) males and were 75.39 years old on average. Comorbidities including cancer, diabetes mellitus, chronic hepatic disease, chronic renal disease and chronic cardiac disease, were evaluated, as shown in Fig. 2A. The total in-hospital mortality was 36.7%. However, as shown in Fig. 2B, the Sepsis-3 (0.514; 95% CI 0.500, 0.528), as well as the two most widely used severity assessment tools in SCAP, PSI (0.550; 95% CI 0.526, 0.575) and CURB-65 (0.580; 95% CI 0.558, 0.602) scores, were poor predictors of in-hospital mortality in elderly SCAP patients in the present study.

The 2365 enrolled patients were divided into a training cohort (1655 patients) and a testing cohort (710 patients). Comparing the two cohorts, we found no significant differences in sex ratio (64.7% vs. 65.1% males, *p* = 0.780), mean age (75.30 vs. 75.31 years old, *p* = 0.971), prognosis, treatment, comorbidities, vital signs on admission, or most laboratory examinations (Table 1).

**Table 1:** Baseline characteristics of elderly SCAP‡ individuals in training cohort and testing cohort

Variables	Overall (*n* = 2365)	training cohort (*n* = 1655)	testing cohort (*n* = 710)	P^†^-value	
Demographic characteristics					
Age (years old)	74 (69, 80)	75 (69, 81)	74 (69, 80)	0.871	
Male, n (%)	1529 (64.7)	1067 (64.5)	462 (65.1)	0.780	
Prognosis					
7-day mortality, n (%)	152 (6.4)	103 (6.2)	49 (6.9)	0.538	
14-day mortality, n (%)	311 (13.2)	213 (12.9)	98 (13.8)	0.538	
28-day mortality, n (%)	621 (26.3)	426 (25.7)	195 (27.5)	0.382	
ICU mortality, n (%)	755 (31.9)	519 (31.4)	236 (33.2)	0.368	
In-hospital mortality, n (%)	868 (36.7)	600 (36.3)	268 (37.7)	0.490	
Length of stay in hospital(d)	21 (12, 31)	21 (12, 31)	20 (12, 31)	0.788	
Treatment					
Vasopressor, n (%)	1432 (60.5)	993 (60.0)	439 (61.8)	0.404	
Comorbidities					
Cancer, n (%)	428 (18.1)	312 (18.9)	116 (16.3)	0.146	
Diabetes mellitus, n (%)	392 (16.6)	271 (16.4)	121 (17.0)	0.689	
Chronic hepatic diseases (%)	65 (2.7)	43 (2.6)	22 (3.1)	0.495	
Chronic renal diseases (%)	175 (7.4)	130 (7.9)	45 (6.3)	0.196	
Chronic cardiac diseases (%)	995 (42.1)	717 (18.5)	278 (39.2)	0.060	
Vital signs on admission					
Respiratory rate(times/min)	19 (14, 23)	19 (14, 24)	18 (14, 23)	0.240	
Heart rate (beat/min)	95.47 (94.51–96.43)	95.8 (94.75–97.03)	94.49 (92.71–96.27)	0.213	
Systolic blood pressure (mmHg)	128.72 (127.38-130.06)	128.62 (126.96-130.18)	129.08 (126.65–131.50)	0.734	
Diastolic blood pressure (mmHg)	69.89 (69.10-70.68)	69.88 (68.93–70.83)	69.90 (68.47–71.34)	0.918	
Laboratory examinations					
White blood cell (× 109/L)	9.75 (6.58, 13.37)	9.53 (6.52, 13.36)	9.70 (6.75, 13.39)	0.3664	
Monocyte (× 10^9^/L)	0.43 (0.27, 0.62)	0.42 (0.27, 0.62)	0.43 (0.27, 0.62)	0.940	
Neutrophil (× 10^9^/L)	7.87 (4.91, 11.53)	7.77 (4.88, 11.50)	8.23 (5.01, 11.82)	0.175	
Lymphocyte (× 10^9^/L)	0.83 (0.53, 1.24)	0.83 (0.53, 1.24)	0.81 (0.53, 1.21)	0.307	
Platelet (× 10^9^/L)	165 (106, 237)	165 (104, 240)	166 (110, 236)	0.699	
Hemoglobin (g/L)	107.95 (106.92-108.98)	107.67 (106.42-108.92)	108.60 (106.76-110.43)	0.441	
Total bilirubin (µmol/L)	11.2 (7.8, 16.6)	11.0 (7.7, 16.1)	11.6 (8.1, 17.6)	0.057	
Direct bilirubin (µmol/L)	5.3 (3.5, 8.4)	5.1 (3.5, 8.1)	5.4 (3.5, 8.8)	0.031	
Albumin (g/L)	32.82 (32.56–33.07)	32.84 (32.53–33.15)	32.76 (32.30-33.23)	0.741	
Globulin (g/L)	25.71 (25.46–25.95)	25.76 (25.46–26.06)	25.58 (25.14–26.01)	0.535	
ALT^‡^ (IU/L)	20 (12, 37)	19 (12, 36)	20 (13, 38)	0.161	
AST^‡^ (IU/L)	27 (19, 47)	27 (19, 46)	29 (20, 51)	0.068	
Creatinine (µmol/L)	75.00 (56.35, 109.00)	75.00 (57.00, 108.15)	75.00 (55.25, 112.00)	0.600	
BUN^‡^ (mg/dL)	7.63 (5.36, 12.30)	7.53 (5.40, 12.40)	7.81 (5.21, 12.13)	0.693	
Lactate (mmol/L)	1.5 (1.1, 2.0)	1.5 (1.1, 2.0)	1.5 (1.1, 2.1)	0.384	
Uric acid (µmol/L)	243.0 (152.9, 346.6)	247.5 (152.3, 349.0)	235.0 (153.8, 337.0)	0.413	
Glucose (mmol/L)	7.12 (5.88, 9.59)	7.13 (5.90, 9.58)	7.09 (5.71, 9.60)	0.640	
APTT^‡^ (s)	32.3 (27.9, 38.9)	32.3 (28.0, 38.8)	32.3 (27.9, 39.8)	0.766	
PT^‡^ (s)	12.9 (11.8, 14.4)	12.9 (11.9, 14.4)	12.8 (11.8, 14.5)	0.688	
D-Dimer (mg/L)	4.53 (2.23, 9.05)	4.62 (2.28, 9.05)	4.37 (2.13, 9.03)	0.582	
Troponin T (ng/mL)	27.90 (19.95, 68.80)	27.45 (20.0, 65.9)	28.80 (19.77, 75.05)	0.301	
BNP‡ (pg/mL)	1078 (553.5, 3939.5)	1088 (564, 4046)	1043 (536, 3790)	0.573	
PCT‡ (ng/mL)	0.33 (0.15, 1.10)	0.33 (0.15, 1.08)	0.33 (0.15, 1.12)	0.195	
IL-6(pg/ml)	47.83(27.70, 102.70)	47.83 (27.61, 102.60)	47.83 (27.82, 104.08)	0.626	
CRP‡ (mg/L)	63.45 (27.55, 105.00)	63.45 (27.20, 104.00)	63.45 (81.10)	0.945	

^†^ Data was shown as median [interquartile range (IQR), 25–75%] or mean [± standard deviation (SD)] for continuous variables, and frequency for categorical variables

^‡^SCAP: severe community-acquired pneumonia; n: number; ALT: alanine aminotransferase; AST: aspartate aminotransferase; BUN: blood urea nitrogen; APTT: activated partial thromboplastin time; PT: prothrombin time; BNP: brain natriuretic peptide; CRP: C-reactive protein; PCT: procalcitonin

### Construction of the nomogram

In the training cohort, 26 variables were found to be potentially significant difference in univariate logistic regression analysis. Next, these variables were reanalyse by multivariate logistic regression, and 6 factors were used as independent risk factors for in-hospital mortality to construct the prediction model, including age, the use of vasopressor, chronic renal disease, neutrophil, platelet, and blood urea nitrogen (BUN). Their ORs and 95% Cis were presented in Table 2.

**Table 2:** Risk factors associated with in-hospital mortality in training cohort

Risk factors	Univariate analysis		Multivariate analysis	
	OR (95% CI)	P ^†^	OR (95% CI)	P
Age	1.049 (1.034, 1.063)	< 0.001	1.042 (1.025, 1.058)	< 0.001
The use of vasopressor	3.939 (3.127, 4.963)	< 0.001	3.748 (2.933, 4.789)	< 0.001
Comorbidities				
Chronic hepatic diseases	2.063 (1.123, 3.788)	0.020		
Chronic renal diseases	3.844 (2.633, 5.613)	< 0.001	3.309 (1.995, 4.631)	< 0.001
Vital signs				
Respiratory rate	1.029 (1.013, 1.045)	< 0.001		
Heart rate	1.009 (1.005, 1.013)	< 0.001		
Systolic blood pressure (mmHg)	0.996 (0.993, 0.999)	0.018		
Diastolic blood pressure (mmHg)	0.992 (0.987, 0.997)	0.003		
FiO_2_	1.011 (1.006, 1.015)	< 0.001		
Laboratory examinations				
Neutrophil (×109/L)	1.043 (1.024, 1.062)	< 0.001	1.021 (1.000, 1.042)	0.050
Lymphocyte (×109/L)	0.789 (0.678, 0.917)	0.002		
Hemoglobin (g/L)	0.991 (0.987, 0.995)	< 0.001		
Platelet (×109/L)	0.997 (0.996, 0.998)	< 0.001	0.998 (0.997, 1.000)	0.005
APTT ^‡^ (s)	1.018 (1.010, 1.025)	< 0.001		
PT^‡^ (s)	1.039 (1.019, 1.059)	< 0.001		
Albumin (g/L)	0.961 (0.946, 0.977)	< 0.001		
ALT^‡^ (IU/L)	1.001 (1.000. 1.001)	0.022		
AST^‡^ (IU/L)	1.001 (1.000, 1.001)	0.015		
Creatinine (µmol/L)	1.002 (1.001, 1.003)	< 0.001		
Uric acid (µmol/L)	1.001 (1.001, 1.002)	< 0.001		
Myoglobin (ng/mL)	1.000 (1.000, 1.001)	< 0.001		
BUN^‡^ (mg/dL)	1.050 (1.036, 1.064)	< 0.001	1.032 (1.009, 1.055)	0.006
Glucose (mmol/L)	1.045 (1.018, 1.074)	0.001		
CRP^‡^ (mg/L)	1.002 (1.001, 1.004)	< 0.001		
Lactate (mmol/L)	1.104 (1.044, 1.167)	< 0.001		
PCT^‡^ (ng/mL)	1.013 (1.003, 1.024)	0.009		

^†^ p value of ≤ 0.05 was considered to be statistically significant

^‡^ APTT: activated partial thromboplastin time; PT: prothrombin time; ALT: alanine aminotransferase; AST: aspartate aminotransferase; BUN: blood urea nitrogen; CRP: C-reactive protein; PCT: procalcitonin

**Fig. 3:**
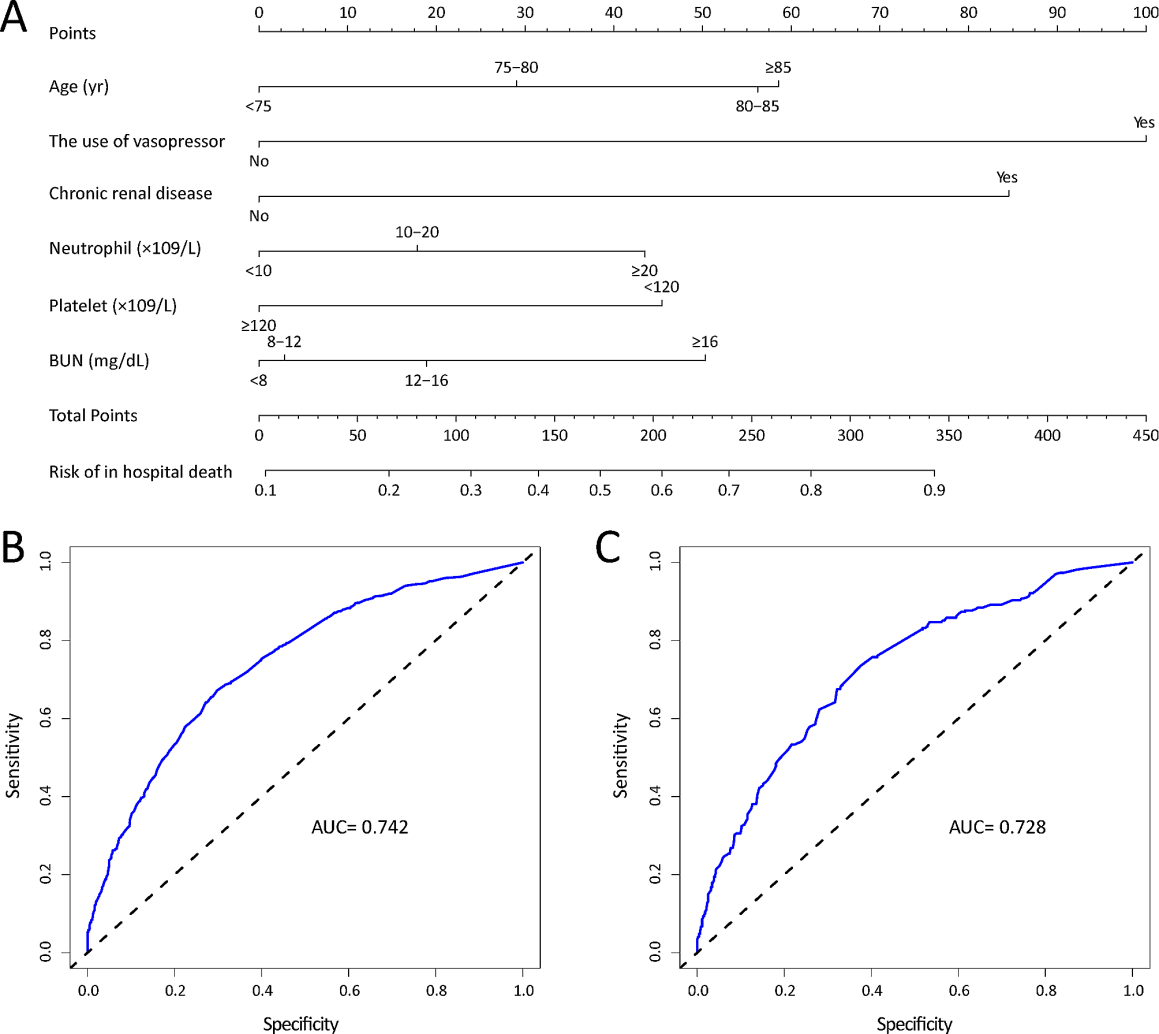
**A** Nomogram for hospital mortality in elderly SCAP patient. **B** The ROC curve of nomogram for training cohort. **C** The ROC curve for testing cohort

The nomogram showed the prediction model for individual hospital morbidity illustrated by the 6 factors above in Fig. 3A. Each variable corresponded to a point on the top line. And the sum of these 6 points corresponded to the “total points” line vertically projected on the risk of in-hospital death on the bottom line.

### Assessment of the nomogram

**Fig. 4:**
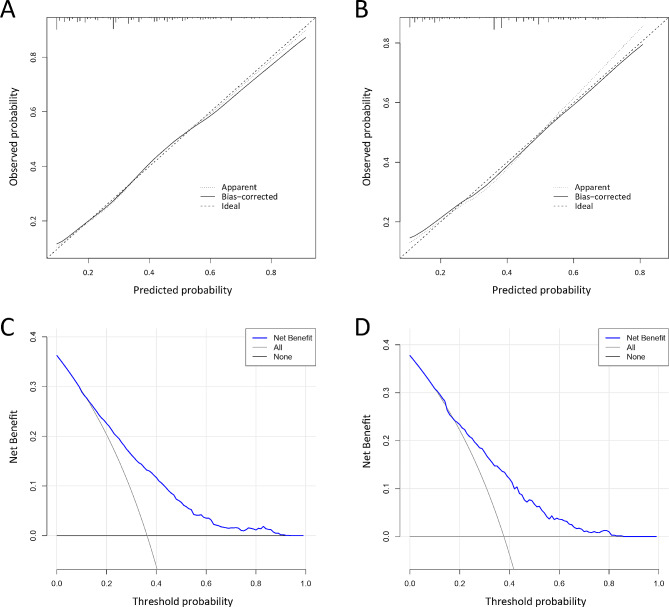
**A** Calibration curve of nomogram in training cohort. **B** Calibration curve of nomogram in testing cohort. **C** The DCA of nomogram for training cohort. **D** The DCA of nomogram for testing cohort

The C index was 0.743 (95% CI 0.719–0.768) in the training cohort and 0.731 (95% CI 0.694–0.768) in the testing cohort, which indicated that the prediction model had good predictive discrimination in elderly SCAP patients. The ROC curves and AUC for the training cohort and testing cohort (AUC = 0.742 vs. 0.728) were displayed in Fig. 3B and C, which indicated a robust discrimination of this prediction model. The calibration plots released a consistency between prediction model probabilities and observed probabilities in Fig. 4A and B. Then, the DCA demonstrated great net benefit across different threshold probabilities, as shown in Fig. 4C and D, possessing strong clinical practicality.

## Discussion

CAP is a significant public health issue associated with substantial morbidity and mortality in all age groups globally [16, 17]. The incidence of CAP in the United States is a significant contributor to hospitalization and mortality, with an estimated annual report of approximately 6 million cases [4, 18]. A surveillance study (*n* = 2488 adults) reported that 21% of CAP patients had progressed to SCAP [19]. The mortality for SCAP patients ranged between 17% and 49% in some large multicenter cohort studies [20–22]. In elderly patients, CAP exhibits the absence of typical symptoms that are commonly observed in younger adults, owing to attenuated local and systemic inflammatory responses [23], making it difficult to notice in a timely manner. Moreover, it was reported that the incidence of SCAP increased significantly with age [19]. With an overall increase in the elderly population, the burden of caring for elderly SCAP patients would be further increased [23]. To determine the association between SCAP and in-hospital mortality in elderly patients and to make more accurate predictions, we analyzed data from a large, well-characterized retrospective study.

Our prediction model was employed to assess the mortality risk in elderly SCAP patients, showing excellent predictive discrimination ability and great robustness. Owing to the easily obtainable data in routine clinical practice, this model offered a reference about prognostic determination in elderly patients with SCAP, thereby assisting healthcare professionals. Song, Y., et al. [24] analyzed the data from 292 elderly patients with SCAP from 33 hospitals in China. They concluded that age (OR 1.138; 95% CI 1.037–1.253), Glasgow score (OR 0.908; 95% CI 0.838–0.985), blood platelet (OR 0.996; 95% CI 0.993–0.999), and BUN values (OR 1.061; 95% CI 1.023–1.102) were found to be significantly associated with 28-day mortality, which was similar to our results. However, it was regrettable that they did not perform internal validation or confirm their model. Pan, J., et al. [25] enrolled 455 patients with SCAP admitted to the ICU and discovered that lymphocytes, PaO2/FiO2, shock, and APACHE II score were independent risk factors for in-hospital mortality. They conducted an external validation for their prediction model, but the mean age in the development cohort and validation cohort was significantly different (*p* = 0.006). Wang, X., et al. [26] reported that serum creatinine, leukocyte, C reactive protein, GCS and serum HCO_3_^−^ were carried out and that each index was an independent factor for hospital mortality in 37,348 SCAP patients in the ICU. They included a large population of SCAP patients and had a great predictability, but not aimed at elderly people, while more detailed elderly age group analysis was always ignored in most researches.

Age was thought to be an important factor that determined mortality. Previous studies showed that the mortality of CAP patients aged 65 or above was higher than that of those younger than 65 years old (10.3 versus 2.2%) [27]. In some studies that only enrolled elderly patients, the conclusion still held [24]. Age was also an independent risk factor in SCAP patients with heart disease [28] and type 2 diabetes [29]. Immunoreaction influences the prognosis. Qiu, Y., et al. [30] reported a higher neutrophil level in the death group in adult renal transplant recipients. Zhu, Y., et al. [31]found that the CD3^+^ CD4^+^ T cell count (OR 0.987; 95% CI 0.983–0.991) was an independent risk factor for mortality. Neutrophilia and lymphopenia were generally considered to be common immune responses during infection, which might be driven by emargination and delayed apoptosis of neutrophils as well as margination and accelerated apoptosis of lymphocytes [32]. Invasive mechanical ventilation was considered to be an important factor associated with high morbidity. Miquel Ferrer et al. [33] verified the hypothesis that invasive mechanical ventilation (OR 3.54; 95% CI 1.45–8.67) was an independent risk factor for death in SCAP patients. They did not limit the range of age, but the average age of the 644 enrolled patients was up to 65 years old. Impaired renal function also significantly affected the survival rate of elderly SCAP patients according to our model.

Fine and colleagues developed the PSI, a composite score consisting of 20 items that are aggregated to categorize patients into one of five risk groups [34]. Another widely employed prognostic score, the CURB-6, is commonly used due to its simplicity in assessment [35], which is widely used to evaluate the prognosis of patients with pneumonia [2]. However, the prognostic evaluation of PSI in the elderly population is not ideal [14, 15]. Naito, T., et al. [36] evaluated the PSI in patients aged 80 and older and found that the specificity was only 15% when defining PSI Class IV and V as a high-risk group. Baek, M.S., et al. [37] evaluated the PSI and CURB-65 in 160 patients aged 80 or older admitted to the medical ICU, but concluded that the performances of the CURB-65 and PSI were not excellent in very elderly patients. Chen, J.H., et al. [38] compared the PSI and CURB-65 categories across three age categories: younger adults (18–64 years), elderly adults (65–84 years) and very old adults (≥ 85 years). The AUCs for the PSI were 0.87, 0.85 and 0.69, respectively, and the AUCs for CURB-65 were 0.80, 0.73 and 0.60 in the three groups. The inappropriate weight given to the age variable was thought to be the reason for underperformance of the PSI in elderly patients. Sepsis-3 has been widely used in ICU patients, and also can be used in prognostic prediction in patients developing sepsis secondary to community-acquired pneumonia [13, 39]. Compared to the PSI, CURB-65 and Sepsis-3, our model focused specifically on elderly SCAP patients aged 65 years or older. The prognosis assessment is more predictive in elderly patients.

The advantage of our study was that we encompassed a large population in both the training and testing cohorts with complete information, thereby enhancing the robustness of our model. Disadvantages were also present. First, we excluded patients with severe immunosuppression, which indicated a significant burden of morbidity. This group of patients with an alternat immune system due to an underlying disease or medical treatment is at elevated risk of pneumonia by uncommon avirulent or opportunistic organisms [40], which may make the prediction model biased differently from the other group of patients. Therefore, we excluded patients with severe immunosuppression. Admittedly, this model is more applicable to non-immunodeficient populations. Then, we did not follow up long enough to record long-term mortality (> 3 months). However, further evaluation was needed to explore long-term mortality factors, incidence, prediction, and their implications for patient care in individuals with SCAP [13]. Several studies have reported that advanced age, sex, comorbidities, pneumonia type, and illness severity are associated with increased long-term mortality risk. Third, while internal verification was conducted, external validation was not achieved. The data collected from a single hospital limited the representativeness of the patient population. Fourth, despite a thorough analysis of potential risk factors, it is essential to know the possibility that some unadjusted confounders and untested variables may enhance the model.

The prognosis of elderly patients with SCAP is influenced by multiple factors. This model has a certain reference role in predicting mortality, and the indicators included in the model are easily available clinical examination results. We hope our model can offer a reference to clinicians in their daily work as a tool to convey prognostic information.

## Conclusion

In conclusion, we constructed a prognosis prediction model for in-hospital mortality in elderly patients with SCAP. The nomogram incorporated six risk factors encompassing age, the use of vasopressor, chronic renal disease, neutrophil, platelet and BUN. It had great predictive accuracy and robustness, while also demonstrating clinical practicality at ICU admission. The assessment could facilitate the early identification of high-risk patients, thereby ensuring that high-risk individuals would receive adequate attention and timely interventions.

## Data Availability

The datasets used and/or analyzed during the current study are available from the corresponding author on reasonable request.

## Abbreviations

Community: Acquired pneumonia (CAP)
Severe community: Acquired pneumonia (SCAP)
ICU: Intensive care unit
PSI: Pneumonia Severity Index
IQR: Interquartile range
SD: Standard deviation
CIs: Confidence intervals
C index: Concordance index
ROC: Receiver-operating characteristic
AUC: Area under curve
DCA: Decision curve analysis
BUN: Blood urea nitrogen
